# Atypical Mal de Meleda in a Hispanic Patient

**DOI:** 10.1155/2023/6640311

**Published:** 2023-09-14

**Authors:** Mónica Guevara, Michelle Mafla, Camila Miño

**Affiliations:** ^1^Dermatology Service, Pablo Arturo Suarez Hospital, Ángel Ludeña y Machala Oe5261, 170702 Quito, Ecuador; ^2^School of Medicine, Pontifical Catholic University of Ecuador, Ave 12 de Octubre 1076, 170143 Quito, Ecuador; ^3^Public Health, London School of Hygiene & Tropical Medicine, Keppel street, WC1E 7HT, London, UK

## Abstract

Mal de Meleda (MDM) is a rare autosomal palmoplantar keratoderma (PPK) skin disorder (estimated incidence of 1 per 100,000 people) commonly associated with consanguinity and early childhood onset. MDM is characterized by bilateral diffusion of PPK plaques with delimited yellowish lesions that transgredien to the dorsum of the hands and feet. Additional features include nail dystrophy, lichenoid lesions, hyperhidrotic maceration, involvement of the knees and elbows, malodor, fungal superinfections, and digital constrictions. A male patient aged 42 years presented with asymptomatic, chronic, and diffused PPK lesions that progressed to the dorsal surface of the hands and feet, along with knees and elbows involvement. On clinical examination, asymmetrical lesions were observed on the hands, the left palm with yellowish waxy hyperkeratotic plaques, and the right palm with erythematous scaling and hyperkeratotic interphalangeal rings. The soles of the feet presented with yellow waxy hyperkeratotic plaques. In addition, nail dystrophy and loss of dermatoglyphics were observed. Initially, symptomatic topical treatment was established. However, owing to the lack of clinical response, a biopsy was performed, which revealed thickened corneal layer, acanthosis, spongiosis, and perivascular lymphohistiocytic infiltrate. MDM diagnosis was confirmed based on a personal history of consanguinity, clinical presentation with absence of systemic symptoms, and transgredien pattern of the lesions. Systemic treatment with low doses of isotretinoin (10 mg orally everyday) was initiated, and two months later, slight clinical improvement has been observed until date. The present case report describes MDM in a Hispanic patient, who presented with asymmetric PPK lesions on the hands and received isotretinoin treatment.

## 1. Introduction

Mal de Meleda (MDM) is a rare autosomal recessive hereditary palmoplantar keratoderma (PPK) caused by mutations in the secreted LY6/PLAUR-related protein 1 (SLURP1) gene, responsible for epidermal homeostasis and wound healing [1, 2]. MDM commonly appears after birth and is characterized by bilateral PPK that progresses to the dorse of hands and feet [3]. No specific treatment for MDM has been established, but oral systemic retinoids have proven to be effective [4, 5]. The current clinical case describes a Hispanic patient with asymmetric MDM who received isotretinoin.

## 2. Case Presentation

A 42-year-old Hispanic male presented with persistent diffuse PPK that transgressed to the dorsum of the hands, feet, wrist, elbows, and knees. At 3 months of age, his mother noticed hyperkeratotic plaques initially on the fingertips of both hands. During early childhood, the lesions spread to the surface of the palms and soles and extended to the dorsal aspects of the hands, feet, ankles, and wrists. Also, the patient complained of simultaneous desquamation and hyperkeratosis accompanied by hyperhidrosis or xerosis on different limbs. He reported itching during environmental heat and absence of tactile, thermal, and painful sensations in the palms and soles, which have caused minor unnoticed injuries. The patient presented a history of consanguinity; his parents were second-degree relatives. None of the siblings or relatives reported similar symptoms.

**Figure 1:**
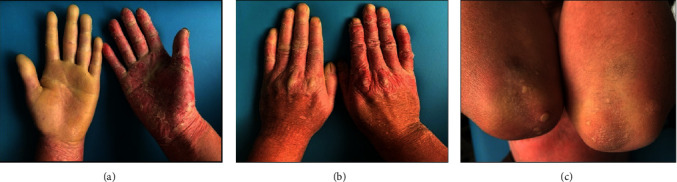
Mal de Meleda clinical features of hands and elbows: (a) hyperkeratotic plaque in left hand and desquamation in right hand, (b) keratoderma on the dorse of hands was limited to proximal phalanges and nail involvement characterized by koilonychia, onycholysis, yellowish chromonychia, and dystrophy, and (c) hyperkeratotic-lichenified plaques on elbows with slight desquamation.

**Figure 2:**
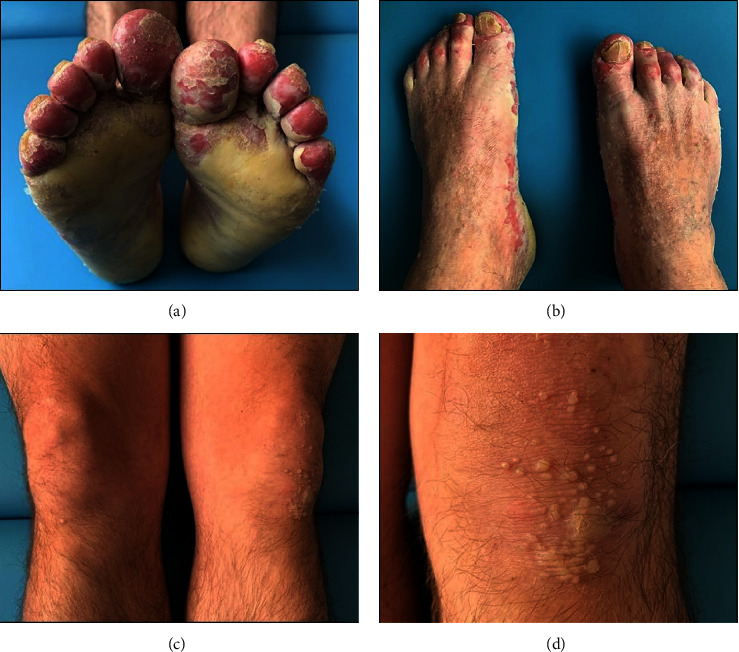
Mal de Meleda clinical features of feet, soles, and knees: (a) Soles with thick, yellowish, and waxy keratoderma and fingertips with desquamation and erythematous background. (b) Scaly hypochromic plaques on feet dorse and nail involvement of both feet. Foot nails with dystrophy, slight onychogryphosis, yellowish chromonychia, and koilonychia. (c) Knees with hyperkeratotic small plaques, scaly points, and loss of body hair. (d) Left knee with hyperkeratotic plaque and slight desquamation.

Clinical examination revealed yellowish waxy hyperkeratotic transgressive plaques on the left palm. Desquamation with erythematous background and interphalangeal hyperkeratotic rings were observed on the right palm. Also, nail involvement was observed in both hands. The elbows had hyperkeratotic lichenified plaques, and fingertips showed loss of dermatoglyphics (Figure 1). The soles presented hyperkeratotic plaques, while the fingertips had desquamation. Interphalangeal and periungual areas showed scaly hypochromic plaques with desquamation in the dorse of the feet. The knees had hyperkeratotic plaques and scaly points, and loss of body hair (Figure 2). The remaining physical examination was normal.

**Figure 3:**
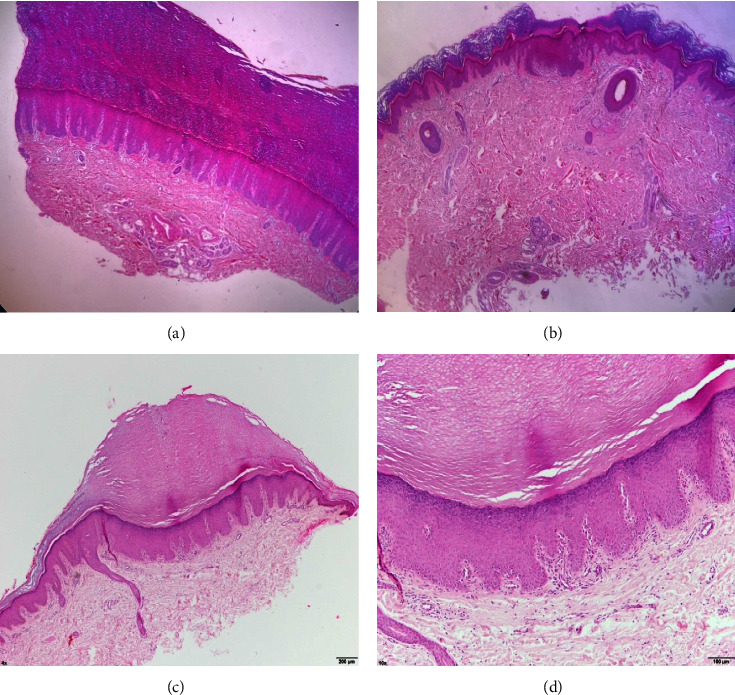
Histopathological images of the patient with hematoxylin and eosin staining: (a) right hand (10×), (b) right knee (10×), (c) right elbow (10×), and (d) right elbow (40×).

The patient was hospitalized approximately 10 years ago to study his case. However, his diagnosis remained uncertain since multiple studies performed were normal and without alterations. Throughout his life, the patient has received steroids, keratolytic agents, and ointments without clinical improvement. However, 15 years ago, during his residence in another country, he was given acitretin (unknown dose) for 3 years and reported overall improvement. One year ago, the patient reported had been diagnosed with palmoplantar pityriasis rubra pilaris (PRP) which was managed with fluconazole (150 mg orally every week) and clotrimazole cream 1% (topically applied every day). PRP was ruled out because the patient did not present coalescing red-orange keratotic follicular plaques [6]. Topical treatment twice a day with Burrow's solution (aluminum triacetate) immersion, clobetasol 0.1% + salicylic acid 20% + lactic acid 2% cream, and urea 5% moisturizing cream was initiated. Fluconazole and clotrimazole cream 1% were suspended. One month later, due to the lack of clinical response, a punch biopsy was performed on the right palm and right knee. Histopathological examination revealed a thickened corneal layer, pronounced stratum lucidum, marked acanthosis, spongiosi, and a prominent perivascular lymphohistiocytic infiltrate. In order to confirm that the lesions on the elbows and knees formed part of the same spectrum of genodermatosis, we performed a punch biopsy on the right elbow. The histological report presented a nonepidermolytic hyperkeratosis characterized by scant lymphocytic infiltrate around the papillary vessels and a thick plug of compact keratin that compressed the epidermis, which presented a thick granular layer and acanthosis with regular hyperplasia of the networks (Figure 3). The laboratory test results were unremarkable.

Diffuse hereditary PPKs include multiple disorders such as Vörner-type, Nagashima-type, Bothnia-type, Greither-type, Gamborg–Nielsen/Norrbotten-type, and MDM [5]. However, the MDM diagnosis was made considering the early disease onset, clinical presentation with absence of systemic symptoms, history of inbreeding, and histopathological findings. The genetic analysis of SLURP1 mutation could not be performed. Isotretinoin at low doses (10 mg orally daily) was initiated with a progressive dose increase to assess tolerance. Two months later, the patient presented clinical improvement with reduced thickness of PPK lesions, diminution of desquamation, and recovery of the tactile and thermal sensation in both hands. Isotretinoin dose was increased to 20 mg orally daily due to the good tolerance and absence of adverse effects till the date of this report.

## 3. Discussion

MDM has an estimated prevalence of 1/100,000 and was first reported on the Croatian island of Meleda (1826) owing to its high risk of inbreeding [1, 7]. Our patient had a history of second-degree consanguinity, as in previous cases [1, 4, 8]. MDM begins in early childhood as palmoplantar erythema lesions, but we reported fingertips hyperkeratotic plaques as initial lesions [9]. The diagnosis for MDM includes clinical signs, histologic findings, and genetic testing of SLURP1 mutations [4, 5]. The clinical characteristics and location of the lesions were consistent with previous cases, nevertheless we described asymmetric lesions on hands (hyperkeratotic left palm and desquamative right palm) [3]. In addition, the presence of hyperkeratotic lichenified plaques on elbows and knees with slight desquamation was unusual with the possibility of a new MDM genetic variant. Previous reports of MDM evidenced psoriasiform plaques on the elbows and knees [7], but only in the case report by Nath et al., the patient showed well-defined lichenoid papules on the extensor aspects of the elbows and knees [10]. Our patient had normal laboratory values, and the histological report was in accordance with the typical findings reported for MDM [9].

The clinical presentation and histological report were incompatible with PRP. It is characterized by painful diffuse red-orange PPK papules, and the histological findings include orthokeratosis or parakeratosis, hypergranulosis, irregular acanthosis, and perivascular lymphohistiocytic dermis infiltrate [6]. The rarity of hereditary PPKs represents a challenge, but the morphological distribution of the lesions guided our diagnosis [4, 5]. Moreover, the absence of extracutaneous or additional symptoms permitted to reject syndromic PPKs. Other differential diagnoses considered were PPK Vörner-type, Nagashima-type, Bothnia-type, Geither-type, and Gamborg–Nielsen/Norrbotten-type [5]. Nevertheless, they were ruled out by differences in the clinical presentation, diffuse transgredien lesions without granular degeneration, and the apparent recessive inheritance pattern [9].

MDM lacks an established therapeutic protocol; nonetheless, some treatments have been better studied than others. General recommendations for the management of PPKs included topical keratolytic agents, dermabrasion, oral retinoids, and treatment of complications [5]. Oral acitretin has been effective in previous MDM reports, but it was not available in our country [4, 8, 11]. Low-dose isotretinoin was chosen for the patient because it has reported favorable outcomes after 3-4 months of therapy in MDM [12]. The patient requires a longer follow-up, but he has reported clinical improvement hitherto.

A main limitation was the absence of the SLURP1 mutation detection because it was inaccessible in our country. Acitretin, the most commonly used treatment for MDM, could not be administered despite the apparent efficacy reported by the patient due to local inability. Another limitation was the slight clinical response due to the short follow-up of isotretinoin treatment. MDM has a low prevalence; therefore, large-scale clinical trials are not possible, and treatment methods with a high-level of evidence have not been established.

This case is reported for its uniqueness in clinical presentation. Although MDM has been described worldwide, few cases have been reported in Hispanic population. To our knowledge, no case of MDM with asymmetric presentation (simultaneous desquamation and hyperkeratosis) has been previously reported. Consequently, in this case report, we present a Hispanic patient with asymmetric MDM to strengthen and extend the understanding of this condition.

## Data Availability

No underlying data were collected or produced in this study.
